# SAHD-10: Development and initial validation of a short version of the Schedule of Attitudes Toward Hastened Death based on a large multinational sample

**DOI:** 10.1017/S1478951524001524

**Published:** 2025-01-14

**Authors:** Kerstin Kremeike, Kathleen Boström, Thomas Dojan, Cristina Monforte-Royo, Barry Rosenfeld, Raymond Voltz, Christian Rietz, Julia Strupp

**Affiliations:** 1 Department of Palliative Medicine, Faculty of Medicine and University Hospital, University of Cologne, Cologne, Germany; 2 Department of Nursing, Faculty of Medicine and Health Science, Universitat Internacional de Catalunya, Barcelona, Spain; 3 Department of Psychiatry and Behavioral Sciences, Memorial Sloan Kettering Cancer Center, New York, USA; 4 Department of Psychology, Fordham University, Bronx, New York, USA; 5 Center for Integrated Oncology Aachen Bonn Cologne Duesseldorf (CIO ABCD), Faculty of Medicine and University Hospital, University of Cologne, Cologne, Germany; 6 Center for Health Services Research, Faculty of Medicine and University Hospital, University of Cologne, Cologne, Germany; 7 Faculty of Educational and Social Sciences, Department of Educational Science, University of Education Heidelberg, Heidelberg, Germany

**Keywords:** Desire to die, wish to hasten death, SAHD, surveys and questionnaires, palliative Care, neoplasms

## Abstract

**Objectives:**

Wishes to hasten death (WTHDs) are common in patients with serious illness. The Schedule of Attitudes Toward Hastened Death (SAHD) is a validated 20-item instrument for measuring WTHD. Two short versions have also been developed based on statistical item selection. However, all existing versions show some limitations with potential for improvement. This study aims to develop and initially validate a theory-driven and statistically sound SAHD short version based on a large multinational sample to advance the WTHD assessment in different countries and with different legislations.

**Methods:**

A 3-step procedure was carried out including (1) theory-driven item selection, (2) exploratory, and (3) confirmatory factor analysis. We used a data set collected between 1998 and 2020 across 3 different countries (Germany, Spain, USA). Participants were *N* = 1156 complete cases (*n* = 181 German, *n* = 101 Spanish and *n* = 874 US) of severely ill adult in- and outpatients. They had to be ≥18 years and give informed consent.

**Results:**

The exploratory factor analysis revealed that 10 of 11 items previously selected theory-driven loaded on either of 2 factors: (1) WTHD and (2) internal locus of control. These factors showed good to excellent reliability according to Cronbach’s α and McDonald’s Ω, as well as an excellent fit of our data as an overall model for the total sample.

**Significance of results:**

The developed SAHD-10 represents a reliable and valid alternative to the SAHD and an efficient means to measure and further investigate a WTHD in cross-cultural clinical and research settings.

## Introduction

Patients receiving palliative care frequently experience a desire to die (Bellido-Pérez et al. 2017). Such desires can be described as phenomena manifesting on a continuum ranging from a low pressure to act (i.e., acceptance of death or tiredness of life) to a high pressure to act (i.e., latent or acute suicidality) (Kremeike et al. 2021).

The “wish to hasten death” (WTHD) designates a well-researched manifestation of desire to die that is usually characterized by a relatively high level of suffering and pressure to act. The international 2016 consensus definition describes the WTHD as “a reaction to suffering, in the context of a life-threatening condition, from which the patient can see no way out other than to accelerate his or her death” (Balaguer et al. 2016). Moreover, the WTHD “may arise in response to one or more factors, including physical symptoms (either present or foreseen), psychological distress (e.g. depression, hopelessness, fears), existential suffering (e.g. loss of meaning in life), or social aspects (e.g. feeling that one is a burden)” (Balaguer et al. 2016).

As WTHD can be burdensome to patients themselves, their relatives, and health-care professionals, its adequate assessment is important for research as well as in clinical practice. In 1999, Rosenfeld et al. developed the Schedule of Attitudes Toward Hastened Death (SAHD) as a validated questionnaire for assessing the WTHD in patients with acquired immunodeficiency syndrome and cancer (Breitbart et al. 2000; Rosenfeld et al. 1999). The SAHD is a 20-item scale and can be self-administered or by proxy.

To date, the SAHD was translated into 6 different languages (French, Greek, German, Korean, Spanish, Mandarin) and different versions have been validated for additional populations such as older patients or those receiving palliative care (Dürst et al. 2020; Galushko et al. 2015; Mystakidou et al. 2004; Rodríguez-Mayoral et al. 2023; SHIM and HAHM 2011; Villavicencio-Chávez et al. 2014; Wang and Lin 2016). As such, the SAHD is currently 1 of 2 most used instruments for the measurement of WTHD in palliative care, the second being the Desire for Death Rating Scale (DDRS) (Chochinov et al. 1995). The SAHD is more widely used in end-of-life research than the DDRS and displays overall adequate psychometric properties with moderate to high reliability (Cronbach’s α = 0.89 [Original], 0.71 [German], 0.89 [Greek], 0.66 [Korean], and 0.92 [Spanish]) (Galushko et al. 2015; Mystakidou et al. 2004; Rosenfeld et al. 1999; SHIM and HAHM 2011; Villavicencio-Chávez et al. 2014). Concurrent and discriminant validity are reported to be equally satisfying with positive significant correlations with relevant physical (e.g., low functional status and high dependency on care) as well as psychic conditions (e.g., depression and anxiety) (Dürst et al. 2020; Galushko et al. 2015; Mystakidou et al. 2004; SHIM and HAHM 2011; Villavicencio-Chávez et al. 2014). Determining the factorial dimensionality of the SAHD remains inconclusive, as validation studies report divergent solutions: The original version by Breitbart et al. (2000); Rosenfeld et al. (1999), the Spanish and the French (SAHD-Senior) validation claim unidimensionality (Dürst et al. 2020; Villavicencio-Chávez et al. 2014), while the German version describes 2 factors with 3 items each (Galushko et al. 2015).

All 6 individually validated versions of the SAHD show various statistical limitations or problematic items, e.g. because of low endorsement and value of discrimination or wording that can be considered imprecise or irritating for patients (Galushko et al. 2015; Rosenfeld et al. 2000; Villavicencio-Chávez et al. 2014). Furthermore, literature suggests that a 20-item questionnaire might be taxing for vulnerable or weakened patients (Galushko et al. 2015; Villavicencio-Chávez et al. 2014). To account for these issues, American and Spanish short versions of the SAHD were developed with a reduced number of items (Kolva et al. 2017; Monforte-Royo et al. 2017). While both report acceptable reliability and validity, their short versions are based on solely statistical item selection procedures using either a 2PL or Rasch model. Arguably, short versions of the SAHD might therefore still benefit from a more thorough theoretical grounding that is not limited to statistical item selection. Furthermore, most of the validation studies report small sample sizes (Dürst et al. 2020; Galushko et al. 2015; Mystakidou et al. 2004; SHIM and HAHM 2011; Villavicencio-Chávez et al. 2014).

In our experience of using the SAHD in clinical practice and research, we see several conceptual difficulties based on health professionals and researcher perspectives. Moreover, some of our study patients regarded the overall length of the questionnaire as tiring and the wording of specific items as upsetting. An example is item 12 “I enjoy my present life, even with my illness, and would not consider ending it” which was deemed as platitudinous and cynical: “‘Enjoy?’ I’m vegetating and every day I lose a piece of myself!”

Therefore, we suggest that a theory-driven and statistically sound short version of the SAHD, validated on basis of a large multinational sample can be an appealing advancement in the assessment of the WTHD. In order to develop a robust version of the SAHD that could be used in different settings, with different patient groups and in different cultural contexts, we decided to use data that already existed and had been collected in different countries.

## Methods

### Data collection

We analyzed a merged dataset that originates from 9 studies and was collected over 2 decades ([at least] 1998–2020) across 3 different countries (Germany, Spain, USA) (Breitbart et al. 2012, 2010, 2015, 2000; Galushko et al. 2015; Kremeike et al. 2018; Rosenfeld et al. 1999, 2011, 2000; Villavicencio-Chávez et al. 2014; Voltz et al. 2022). These encompass the original studies by Rosenfeld et al. and Breitbart et al. that introduced and validated the first version of the SAHD in the USA (Rosenfeld et al. 1999, 2000), the verbatim German (SAHD-D) and Spanish (SAHD-SV) translation and validation studies (Galushko et al. 2015; Villavicencio-Chávez et al. 2014) as well as 5 studies that applied these validated versions in the field (Breitbart et al. 2012, 2010, 2015; Kremeike et al. 2018; Rosenfeld et al. 2011; Voltz et al. 2022). Formal inclusion criteria for almost all studies comprised age (≥18 years), fluency in the administered questionnaire language (English (SAHD), German (SAHD-D), Spanish (SAHD-SV)), adequate clinical situation (e.g., no cognitive impairment), a palliative diagnosis (e.g. terminal cancer), and (for all studies) written informed consent.

The SAHD, SAHD-D, and SAHD-SV each consist of the same 20 dichotomous items in English, German, or Spanish, respectively. They all employ a true-or-false format with higher scores indicating greater levels of WTHD. The highest score is 20 with items 1, 7, 12, 15, 19, and 20 being reverse-coded. Studies that were conducted in the USA had patients fill the questionnaire in themselves (self-report), whereas the German and Spanish studies had researchers visit patients on-site and administer the questionnaire in face-to-face interviews. The German research team additionally extracted (unpublished) field notes (of patient opinions) on SAHD characteristics.

We merged *N* = 1934 cases of 3 countries (Germany, Spain, USA) into a total sample after thorough consistency testing within subsamples. Information on all 20 items without missing values was available in *N* = 1156 cases; these were used for further data analysis. For information on location, method of assessment or reason for study size as well as handling of missing data, please refer to the original publications (Breitbart et al. 2012, 2010, 2015, 2000; Galushko et al. 2015; Kremeike et al. 2018; Rosenfeld et al. 1999, 2011, 2000; Villavicencio-Chávez et al. 2014; Voltz et al. 2022).

### Analysis

**Figure 1.**
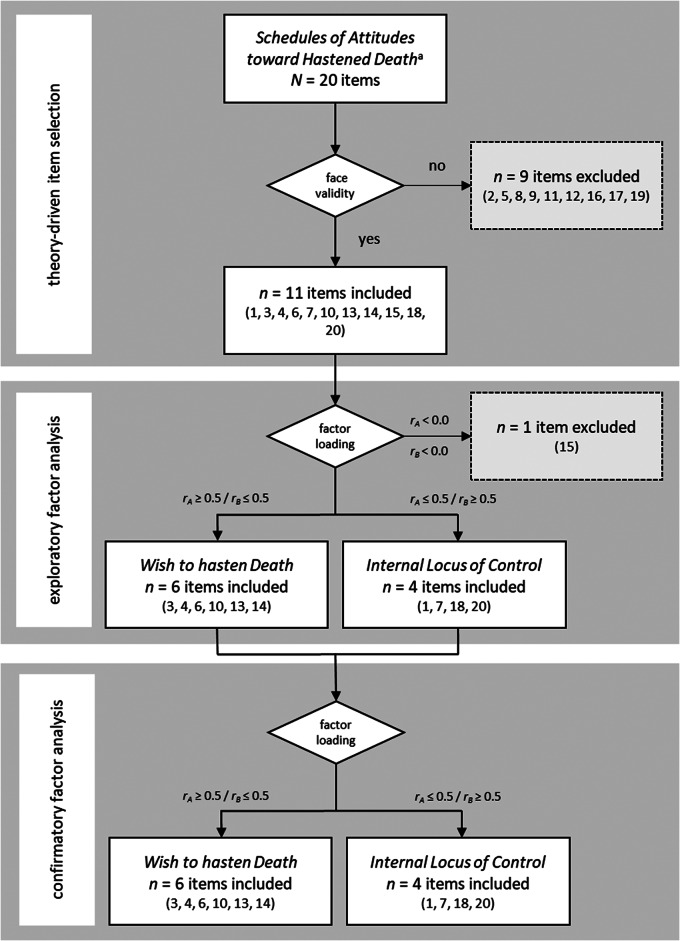
Flowchart of development and initial validation process.

^a^Rosenfeld et al. (1999), Galushko & Strupp et al. (2015), Villavicencio-Chávez et al. (2014).

For the validation and subsequent adaptation of the SAHD, a 3-step procedure was carried out consisting of (1) a theory-driven item selection, (2) an exploratory factor analysis (EFA), and (3) a confirmatory factor analysis (CFA) (see Fig. 1). Through this innovative approach we combine inductive (theoretical item-selection) and deductive methods (EFA based on the assumption of 2 factors) and test findings through CFA.

(1) Theory-driven item selection: To identify adequate and eliminate problematic items, a first round of item selection was conducted following 4 criteria:

- Comparison of item psychometrics from known SAHD validation studies
- Reflection on theoretical fit of items with the WTHD concept (components and predictors)
- Evaluation of patients’ opinions from field notes taken during studies using the SAHD (Kremeike et al. 2018; Strupp et al. 2016; Voltz et al. 2022)
- Estimation of face validity according to experts (KB, TD, KK, JS)

Field notes (step [c]) in form of verbatim transcriptions of patient comments deemed negative or irritated were written down manually during questionnaire administration, transferred into a digital document and summed up for each SAHD-item. Items were then ranked regarding least to most negative comments (see also Table 1).

**Table 1.** Theory-driven item selection

SAHD Items (T = true/F = false)^a^	Validation studies reporting item-specific information	Studies indicating conceptual fit of items	Number of irritation reported in field notes^b^	Decision & rationale
**1.** I feel confident that I will be able to cope with the emotional stress of my illness. (F)	n/a	Monforte-Royo et al. 2012 Rodríguez-Prat et al. 2017	3	**Kept**. Conceptually meaningful (internal locus of control as protective factor against WTHD).
**3.** My illness has drained me so much that I do not want to go on living. (T)	n/a	Rodriguez-Prat et al. 2017	3	**Kept**. Conceptually meaningful (WTHD as exit option).
**4.** I am seriously considering asking my doctor for help in ending my life. (T)	Rosenfeld et al. 2000	n/a	0	**Kept**. Low endorsement but conceptually meaningful (WTHD by request for assisted suicide).
**6.** Dying seems like the best way to relieve the pain and discomfort my illness causes. (T)	n/a	Rodriguez-Prat et al. 2017	2	**Kept**. Conceptually meaningful (WTHD as last option to escape suffering).
**7.** Despite my illness, my life still has purpose and meaning. (F)	Galushko & Strupp et al. 2015	Monforte-Royo et al. 2012 Guerrero-Torrelles et al. 2017 Rodriguez-Prat et al. 2017	0	**Kept**. Low endorsement but conceptually meaningful (‘purpose and meaning’ as protective factors).
**10.** I hope my disease will progress rapidly because I would prefer to die rather than continue living with this illness. (T)	n/a	Monforte-Royo et al. 2017	0	**Kept**. Conceptually meaningful (WTDH as reaction to unbearable disease).
**13.** Because my illness cannot be cured, I would prefer to die sooner, rather than later. (T)	n/a	Monforte-Royo et al. 2017	0	**Kept**. Conceptually meaningful (WTDH as reaction to unbearable disease).
**14.** Dying seems like the best way to relieve the emotional suffering my illness causes. (T)	Rosenfeld et al. 2000	Rodriguez-Prat et al. 2017	2	**Kept**. Highly endorsed and conceptually meaningful (WTHD as exit option).
**18.** I plan to end my own life when my illness becomes too much to bear. (T)	Rosenfeld et al. 2000 Galushko et al. 2015	Monforte-Royo et al. 2012 Rodriguez-Prat et al. 2017	4	**Kept**. Low endorsement & value of discrimination but conceptually meaningful (WTHD as exit option).
**20.** I am able to cope with the symptoms of my illness, and have no thoughts of ending my life. (F)	n/a	Monforte-Royo et al. 2012 Rodriguez-Prat et al. 2017	2	**Kept**. Conceptually meaningful (internal locus of control as protective factor against WTHD).
**15.** Doctors will be able to relieve most of the discomfort my illness causes. (F)	Rosenfeld et al. 2000 Galushko et al. 2015	Rodriguez-Prat et al. 2017	1	**Kept**.^c^ Low endorsement but conceptually meaningful (external locus of control as protective factor).
**2.** I expect to suffer a great deal from emotional problems in the future because of my illness. (T)	Villavicencio-Chávez et al. 2014 Galushko et al. 2015	Rodriguez-Prat et al. 2017	3	**Deleted**. Highly endorsed, but low value of discrimination and conceptually imprecise.
**5.** Unless my illness improves, I will consider taking steps to end my life. (T)	Rosenfeld et al. 2000 Galushko et al. 2015	Rodriguez-Prat et al. 2017	1	**Deleted**. Low endorsement and conceptually imprecise (progressive illness does not ‘improve’).
**8.** I am careless about my treatment because I want to let the disease run its course. (T)	Rosenfeld et al. 2000	n/a	10	**Deleted**. Low endorsement and partly upsetting for patients (patients do not view themselves as ‘careless’).
**9.** I want to continue living no matter how much pain or suffering my disease causes. (F)	Rosenfeld et al. 2000	n/a	7	**Deleted**. Highly endorsed, but partly upsetting for patients (‘no matter how’ wording is irritating).
**11.** I have stopped treatment for my illness because I would prefer to let the disease run its course. (T)	n/a	Monforte-Royo et al. 2017	2	**Deleted**. Conceptually imprecise (‘acceptance of death’ ≠ WTHD).
**12.** I enjoy my present life, even with my illness, and would not consider ending it. (F)	Galushko et al. 2015	n/a	4	**Deleted**. Low endorsement and upsetting for patients (no ‘enjoyment’ of progressive illness)
**16.** Because of my illness, the idea of dying seems comforting. (T)	Rosenfeld et al. 2000	Rodriguez-Prat et al. 2017	6	**Deleted**. Highly endorsed, but partly upsetting for patients (‘comforting’ wording is irritating).
**17.** I expect to suffer a great deal from physical problems in the future because of my illness. (T)	Rosenfeld et al. 2000 Villavicencio-Chávez et al. 2014 Galushko et al. 2015	Guerrero-Torrelles et al. 2017 Rodriguez-Prat et al. 2017	0	**Deleted**. Highly endorsed, but low value of discrimination and conceptually imprecise.
**19.** I am aggressively pursuing all possible treatments because I’ll do anything possible to continue living. (F)	Galushko et al. 2015	Voltz et al. 2010	7	**Deleted**. Low value of discrimination and conceptually imprecise (‘will to live’ and WTHD can coexist).

White background text indicates items kept for statistical analysis, grey background text indicates items deleted after theory-driven item selection (dark grey) or exploratory factor analysis (light grey).

a Positive answers indicated by parentheses.

b Unpublished data by Strupp et al. (2016) and Voltz et al. (2022): number of documented remarks on patient irritation per item.

c Item reexamined and deleted after exploratory factor analysis.

Only items that met all criteria from steps (a) to (d) in a final expert consensus were selected for statistical analysis.

(2) EFA (Mardia et al. 1979): We used principal component analysis (PCA) with a subsequent varimax rotation for models with 1 to 4 factors.

(3) CFA (Beaujean 2014): The subsequent CFA was performed using the R-package “Lavaan.” We used a DWLS-estimation (Diagonally Weighted Least Squares) for categorical or dichotomous variables.

As we are using secondary data from already published studies, we adhered to the reporting recommendations by Streiner and Kottner (2014).

## Results

### Sample

Our multinational sample had a total of *N* = 1156 complete cases, with *n* = 181 from Germany, *n* = 101 from Spain, and *n* = 874 from the USA. German patients came from 2 studies with data collection from 2007–2009 and 2018–2020 (Galushko et al. 2015; Kremeike et al. 2018; Voltz et al. 2022). Diagnoses included terminal cancer, multiple sclerosis, chronic obstructive pulmonary disease, and geriatric multi-morbidity. Patients were included if they were ≥18 years, German-speaking, receiving palliative care, and had no cognitive impairment. The Spanish study included *N* = 101 patients with terminal cancer from 2010–2012 (Villavicencio-Chávez et al. 2014). US patients were included from 5 studies with data collection spanning from 1998–2013. For all further detail on recruitment, study inclusion and patient characteristics, please refer to the original publications (Breitbart et al. 2012, 2010, 2015, 2000; Rosenfeld et al. 1999, 2011).

### First round of item selection according to expert consensus

For the theory-driven item selection, 1 researcher (KB) evaluated each item of the SAHD individually referencing

- relevant literature on psychometric properties of the SAHD and SAHD-D (Galushko et al. 2015; Rosenfeld et al. 2000; Villavicencio-Chávez et al. 2014),
- recent literature on theoretical background of the WTHD construct (Guerrero-Torrelles et al. 2017; Monforte-Royo et al. 2017, 2012; Rodriguez-Prat et al. 2017),
- yet unpublished field notes and think-alouds taken with the aim of assessing feasibility in previous clinical studies that applied the SAHD-D, including opinions of patients who answered the questionnaire (Strupp et al. 2016; Voltz et al. 2022, 2011).

For (c), notes were made by JS, KB, KK, and other members of the respective research teams and then summarized for each item by KB for the present paper. Then, KK, KB, and TD discussed item inclusion based on 2 criteria: (1) the number of field notes expressing patient irritation per item and (2) the content of field notes in form of either emotional upset or issues with comprehension.

Discussing this assessment with 4 experts (KK, KB, TD, CR) yielded consensus on which items properly assess WTHD according to face validity and which do not. The expert group thus agreed on a reduction of the item pool from 20 to 11 items (1, 3, 4, 6, 7, 10, 13, 14, 15, 18, 20). Consulting a fifth expert (JS) confirmed the consensus previously established. For the rationale behind our theory-driven item selection, see Table 1.

### Second round of item selection according to exploratory factor analysis

The EFA revealed that 10 of the 11 selected items load on either of 2 factors: (1) WTHD (3, 4, 6, 10, 13, 14) and (2) internal locus of control (1, 7, 18, 20), see Table 2.

**Table 2.** Exploratory factor analysis using PCA/varimax rotation

	Total *N* = 1156		Germany *n* = 181		Spain *n* = 101		USA *n* = 874	
Factor	**1**	**2**	**1**	**2**	**1**	**2**	**1**	**2**
Item 3	**0.71**	0.29	**0.86**	0.14	**0.47**	0.58	**0.63**	0.39
Item 4	**0.43**	0.52	**0.31**	0.63	**0.67**	0.56	**0.37**	0.48
Item 6	**0.73**	0.24	**0.72**	0.18	**0.77**	0.13	**0.72**	0.26
Item 10	**0.82**	0.08	**0.76**	0.09	**0.69**	0.46	**0.83**	0.08
Item 13	**0.85**	0.10	**0.81**	0.02	**0.87**	0.11	**0.86**	0.09
Item 14	**0.77**	0.15	**0.74**	0.04	**0.80**	0.31	**0.77**	0.15
Item 1	0.04	**0.66**	0.08	**0.62**	−0.05	**0.84**	0.06	**0.65**
Item 7	0.45	**0.38**	0.70	**0.18**	0.69	**0.02**	0.33	**0.49**
Item 18	0.07	**0.71**	−0.04	**0.79**	0.58	**0.21**	0.00	**0.69**
Item 20	0.37	**0.66**	0.59	**0.54**	0.79	**0.14**	0.24	**0.69**
Variance explained	35.86%	19.75%	39.64%	18.19%	45.85%	17.65%	32.25%	20.99%

Bolded numbers do not indicate empirical assignment but reflects theoretical considerations.

The 1 item (15) that did not fit the 2 factorial model was reexamined and excluded from further analysis by theoretical rationale as the item instructs the interviewee to assess their health condition from an external perspective, whereas all other items ask for assessments from their own internal perspective. The EFA thus established the hypothesis that factor 1 exclusively groups items that describe the WTHD, whereas factor 2 exclusively groups items that administer the internal locus of control.

For reliability analysis, we calculated Cronbach’s α and McDonalds Ω to assess internal consistency. The SAHD-10’s internal consistency is satisfying for both factors and in the total (Cronbach’s α: factor 1 = .85, factor 2 = 0.60; McDonalds Omega factor 1 = 0.86; factor 2 = 0.61) as well as the German (Cronbach’s α: factor 1 = .84, factor 2 = 0.59; McDonalds Ω factor 1 = 0.85;, factor 2 = 0.55), Spanish (Cronbach’s α: factor 1 = 0.90, factor 2 = 0.66; McDonalds Ω factor 1 = 0.90;, factor 2 = 0.71) and US subsample (Cronbach’s α: factor 1 = 0.84, factor 2 = 0.59; McDonalds Ω factor 1 = 0.85;, factor 2 = 0.59). Regarding descriptive item statistics, mean, difficulty, standard deviation, and (corrected) selectivity (discriminatory power) had robust values, see Table 3.

**Table 3.** KTT item statistics

		Total (*N* = 1156)			Germany (*n* = 181)			Spain (*n* = 101)			USA (*n* = 874)		
Factor	Item	Mean/ Difficulty	SD	Selectivity^a^	Mean/ Difficulty	SD	Selectivity^a^	Mean/ Difficulty	SD	Selectivity^a^	Mean/ Difficulty	SD	Selectivity^a^
1	3	0.11	0.31	0.64	0.15	0.36	0.75	0.21	0.41	0.63	0.09	0.28	0.60
	4	0.06	0.25	0.47	0.12	0.32	0.37	0.18	0.37	0.77	0.04	0.20	0.41
	6	0.2	0.40	0.66	0.31	0.46	0.66	0.35	0.48	0.67	0.16	0.37	0.65
	10	0.13	0.33	0.69	0.19	0.39	0.62	0.21	0.41	0.79	0.11	0.31	0.68
	13	0.15	0.35	0.74	0.19	0.39	0.75	0.25	0.43	0.77	0.13	0.33	0.73
	14	0.17	0.37	0.67	0.22	0.41	0.61	0.28	0.45	0.78	0.14	0.35	0.66
2	1	0.17	0.37	0.32	0.16	0.37	0.34	0.21	0.41	0.22	0.17	0.37	0.35
	7	0.08	0.27	0.35	0.07	0.26	0.31	0.16	0.37	0.44	0.07	0.26	0.34
	18	0.15	0.36	0.35	0.34	0.47	0.30	0.35	0.48	0.51	0.10	0.30	0.34
	20	0.13	0.34	0.49	0.14	0.35	0.50	0.21	0.41	0.63	0.11	0.31	0.46

a Corrected.

### Confirmatory factor analysis

**Figure 2.**
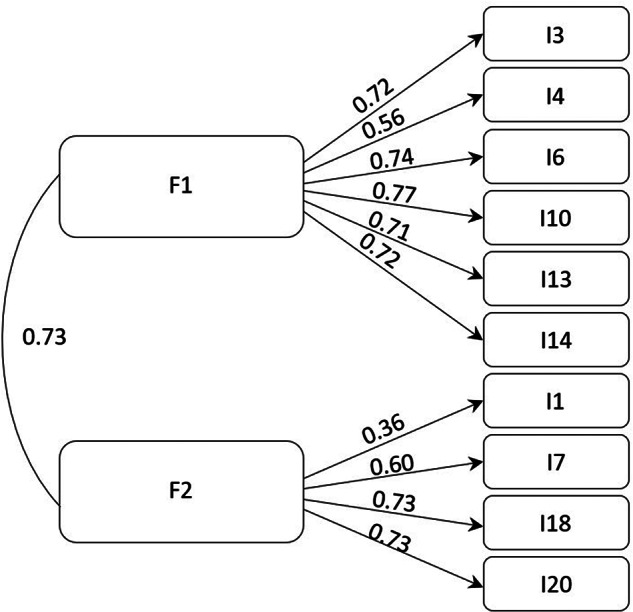
Factor loadings of the total sample.

The 2 factors identified through EFA showed robust factor loadings in the subsequent CFA, see Fig. 2 for the total sample (*N* = 1156).

This was true for the 3 national subsamples as well, as is reported in Table 4.

**Table 4.** Confirmatory factor analysis

	Structural invariance (multigroup comparison)					
	Germany (*n* = 181)		Spain (*n* = 101)		USA (*n* = 874)	
Item	Factor 1	Factor 2	Factor 1	Factor 2	Factor 1	Factor 2
3	0.83		0.61		0.71	
4	0.46		0.81		0.49	
6	0.71		0.74		0.74	
10	0.68		0.79		0.69	
13	0.82		0.84		0.75	
14	0.66		0.86		0.71	
1		0.29		0.27		0.43
7		0.68		0.63		0.58
18		0.24		0.62		0.38
20		0.84		0.83		0.66
*r* Factor		0.80		0.86		0.66

The CFA results show an excellent fit of our data as an overall model for the total sample, confirmed by chi-square goodness-of-fit testing (χ^2^ = 56.28; *df* = 34, *p* = 0.01) as well as CFI = 0.99; RMSEA = 0.02 and SRMR = 0.05. Multi group comparison testing for structural invariance reveals a very good fit for all 3 countries according to chi-square goodness-of-fit testing (χ^2^ = 93.72; *df* = 102, *p* = 0.71) as well as other indicators CFI > .99, RMSEA < .01, and SRMR = 0.06.

## Discussion

Using a 3-stage procedure, we developed and initially validated a theory-based and statistically sound short version of the SAHD (SAHD-10). Our results are based on a large multinational sample of adult in- and outpatients suffering from various severe illnesses. The SAHD-10 comprises items that can be assigned to either factor 1 “desire to hasten death” or factor 2 “internal locus of control.”

When refining and validating existing instruments for other languages or populations and for short versions, decisions on item selection and quality usually are based on statistical properties (Mohamad Adam et al. 2022). Item wording and content is normally only considered regarding appropriateness for other populations or in translations (Epstein et al. 2015). The theory-driven approach preceding the factor analytic validation of the SAHD-10 allowed a reexamination of the existing SAHD on single item-level for conceptual meaningfulness, value of discrimination, level of endorsement and potential to upset patients. Therewith, we ensured that only those items of clinical practicability and relevance remain within the SAHD short version.

Moreover, the SAHD-10 shows very good psychometric properties and internal consistency, both in the total sample as well as in the 3 subsamples. Considering the factorial dimensionality of the SAHD (unidimensionality [Monforte-Royo et al. 2017; Rosenfeld et al. 2000; Villavicencio-Chávez et al. 2014] versus multidimensionality [Galushko et al. 2015]), we found that the SAHD is an instrument with 2 different factors. This is in contrast with most other SAHD-studies which confirmed a unidimensional structure (Monforte-Royo et al. 2017; Rosenfeld et al. 2000; Villavicencio-Chávez et al. 2014).

Numerous SAHD-studies discuss recommendations for potential cutoff scores that allow to discern clinical relevance of SAHD ratings (Kolva et al. 2017; Monforte-Royo et al. 2017; Rosenfeld et al. 2000). It should be discussed whether a number-based sum score can cover the complexity of WTHD since individual attitudes toward hastened death can still vary significantly within acute and non-acute situations (Leitlinienprogramm Onkologie 2020). The in-depth examination on single item level for theory-driven selection revealed that not all items of the SAHD-10’s factor 1 are equally expressive of a suicidal pressure. Therefore, they should not be regarded as indicating equal levels of clinical urgency. Instead, we suggest that examining patient responses on content-level of individual items better serves to identify the type of WTHD. In doing so, the SAHD-10 can provide a basis for addressing the patient’s WTHD – or desire to die – through open, proactive discussion (Voltz et al. 2022).

### Two-dimensional structure of the SAHD-10 and relation to desire to die

In contrast to the definition of WTHD used throughout this paper (Balaguer et al. 2016), the “desire to die” conception used within the German Guideline Palliative Care for Patients with Incurable Cancer provides a broader understanding of the phenomenon (Leitlinienprogramm Onkologie 2020). According to their definition, desires to die manifest on a spectrum ranging from the acceptance of death throughout the desire to die soon and WTHD up to suicidality. These different forms of desire to die are spread across a continuum of increasing suicidal pressure, summarizing several existing models (Balaguer et al. 2016; Lindner 2006; Nissim et al. 2009; Wolfersdorf 2008, 2012; Wolfersdorf and Etzersdorfer 2011).

Research using the SAHD often argues to measure patients’ WTHD according to the international consensus definition (Balaguer et al. 2016; Bellido-Perez et al. 2018; Villavicencio-Chávez et al. 2014). In view of our theory-driven reassessment, we would like to argue that factor 1 of our SAHD-10 covers not only WTHD but also a broader part of the desire to die spectrum. Concerning WTHD, most but not all items of the SAHD-10 tap into some aspect thereof. Items 6 and 14 are expressive of “hypothetically considering hastening death,” whereas items 4 and 13 express an acute “will to die” (including explicit requests for assisted suicide) (Ohnsorge et al. 2014). Items 3 and 10 however do not appear to be expressive of either hypothetical or acute WTHD but rather seem to assess “hoping that dying happens more quickly” (Ohnsorge et al. 2014) and perhaps also “despair” according to Nissim et al. (2009). The SAHD-10 thus appears to be a more fine-grained instrument that is expressive of more aspects of desire to die than just a WTHD.

Factor 2 of our SAHD-10 groups items we propose are expressive of a patient’s internal locus of control, e.g. their belief that they can control their own lives (Rotter 1966). Items 1 and 20 affirm the patient’s ability to cope with the physical and psychological symptoms of their illness. Item 18 displays how even a desire to die itself serve as a form of control for a patient, e.g. by remaining agents of their life through planning to end their life themselves on condition that their illness becomes unbearable (Coyle and Sculco 2004; Royal College of Nursing 2011). The perceived loss of control when confronted with the outlook on disease progression of a life-limiting illness characterized by increasing loss of function is a major risk factor for developing a WTHD (Monforte-Royo et al. 2018). The WTHD and requests for assisted suicide can function as a form of regaining the feeling of control which is central to modern ideas of individualism and agency (Young et al. 2021), as described by item 18. Item 7 expresses that a patient’s life “has purpose and meaning,” a potential protective factor against hopelessness which has been identified as a risk factor of WTHD (Rodin et al. 2009).

When considering influencing factors on a desire to die, we suggest that the measurement of the WTHD should always be combined with a measurement of the will to live. Although there is research considering the will to live a factor opposing a WTHD (Chochinov et al. 1999), other research challenges this notion by pointing out that desire to die and will to live can and do coexist (Voltz et al. 2010). Since the will to live can greatly affect survival and may fluctuate together with desire to die in all stages of disease (Karppinen et al. 2012; Tataryn and Max Chochinov 2002), it can therefore provide important additional information on acuteness of a patients’ WTHD.

This ties into our general recommendation for using the SAHD-10 in the clinical setting. In a first step, the SAHD-10 can be used as a screening tool. A subsequent conversation proactively initiated by the health professional then allows a deeper exploration of the patient’s WTHD. In this case, topics covered by the items of the SAHD-10 can serve as a “door-opener” for meaningful conversation on patient-relevant wishes or fears regarding the end-of-life (Boström etal. 2024a, 2024).

### Strengths and limitations

We present results with several notable strengths: For the development of the SAHD-10, we used an innovative approach that considered various perspectives: (1) international experts on desire to die, (2) development and application of measurement instruments, and (3) patients receiving palliative care. By pooling this expertise and drawing from rich data of different countries and time points for secondary analysis, we also meet criteria of sustainability.

Furthermore, our exceptional data set presents the strengths of a large sample size which allows for a robust factor analysis and yielded a good quantitative analysis. The multinational sample, the long time frame of data collection and the various settings all indicate a heterogenous sample and contribute to the generalizability of our results. Additionally, our combination of inductive and deductive methods with the fundamental theory-driven approach also satisfies qualitative research criteria by not relying on statistical analysis alone.

By proposing a new short version, we also face limitations. First, it is noteworthy that items may have differential potential to upset patients depending on a range of cultural factors (including translation and content). Our sample, while multinational, still reflects a pre-dominantly Western perspective (with US patients weighing for about 80% of the total sample) and may not be able to account for other global regions (Rad et al. 2018). Second, there are translation or cultural issues that cannot be disentangled from our analyses. In our theory-driven approach, we can only present data on irritation through tone or wording of items from the German sample (see Table 1), as field notes were taken only in the context of German studies. Therefore, we are not able to report on US and Spanish patients’ perspectives on the same items. It might be possible that in the US and Spanish language versions, patients could find other items irritating.

There is also no research available yet on the SAHD-10’s discriminant or concurrent validity as well as its use for patients without life-threatening conditions. Further research that tests the SAHD-10 in different samples and alongside other questionnaires assessing relevant related constructs is warranted.

Lastly, one of the strengths of the study was the collaboration with different research teams from the USA, Spain, and Germany. Working in a common cause (even without funding) shows the high motivation of the researchers to continue research on WTHD. As civic and legislative changes on a gobal scale shift toward more liberal attitudes regarding (medical) aid in dying, dealing with the WTHD and successive requests for (medical) aid in dying is becoming more urgent (British Medical Association, 2021). To address the rising attention on the phenomenon of WTHD subsequent to these changes, highly motivated, international collaborative research is therefore of highest importance.

## Supporting information

Kremeike et al. supplementary material Kremeike et al. supplementary material

## Data Availability

Data cannot be shared publicly because participants were guaranteed protection of personal data when giving their consent to participate in the respective studies. For research purposes, data can be shared with individuals only upon reasonable request.

## Supplementary material

The supplementary material for this article can be found at http://dx.doi.org/S1478951524001524.
