# SAHD-10: Development and initial validation of a short version of the Schedule of Attitudes Toward Hastened Death based on a large multinational sample – CORRIGENDUM

**DOI:** 10.1017/S1478951525000410

**Published:** 2025-06-09

**Authors:** Kerstin Kremeike, Kathleen Boström, Thomas Dojan, Cristina Monforte-Royo, Barry Rosenfeld, Raymond Voltz, Christian Rietz, Julia Strupp

**Keywords:** Desire to die, wish to hasten death, SAHD, surveys and questionnaires, palliative care, neoplasms, corrigendum

The Authors apologise for a misspelling in one author name. Cristina Monforte-Royo was previously Cristina Montforte-Royo, but this has been corrected in the published article.
