# Supplementary material for: SAHD-10: Development and initial validation of a short version of the Schedule of Attitudes Toward Hastened Death based on a large multinational sample
Source: Palliat Support Care. 2025 Jan 14;23:e14. doi: 10.1017/S1478951524001524 (PMC13166292; doi:10.1017/S1478951524001524)
Supplement: Kremeike et al. supplementary material [file S1478951524001524sup001.docx]

**Supplement 1:**

**SAHD-10**

| **Nr** | **Item** | **Value** |
| --- | --- | --- |
| **1** | I feel confident that I will be able to cope with the emotional stress of my illness. | **□** true **□** false |
| **2** | My illness has drained me so much that I do not want to go on living. | **□** true **□** false |
| **3** | I am seriously considering asking my doctor for help in ending my life. | **□** true **□** false |
| **4** | Dying seems like the best way to relieve the pain and discomfort my illness causes. | **□** true **□** false |
| **5** | Despite my illness, my life still has purpose and meaning. | **□** true **□** false |
| **6** | I hope my disease will progress rapidly because I would prefer to die rather than continue living with this illness. | **□** true **□** false |
| **7** | Because my illness cannot be cured, I would prefer to die sooner, rather than later. | **□** true **□** false |
| **8** | Dying seems like the best way to relieve the emotional suffering my illness causes. | **□** true **□** false |
| **9** | I plan to end my own life when my illness becomes too much to bear. | **□** true **□** false |
| **10** | I am able to cope with the symptoms of my illness and have no thoughts of ending my life. | **□** true **□** false |
